# Effectiveness of experimental and commercial pertussis vaccines in the elimination of *Bordetella pertussis* isolates with different genetic profiles in murine model

**DOI:** 10.1007/s00430-021-00718-1

**Published:** 2021-08-02

**Authors:** Marta Prygiel, Ewa Mosiej, Karol Wdowiak, Paulina Górska, Maciej Polak, Klaudia Lis, Katarzyna Krysztopa-Grzybowska, Aleksandra Anna Zasada

**Affiliations:** Department of Vaccines and Sera Evaluation, National Institute of Public Health, National Institute of Hygiene, Warsaw, Poland

**Keywords:** Pertussis, Vaccines, Waning immunity, Genetic diversity

## Abstract

The aim of this study was to compare the elimination of *Bordetella* *pertussis* clinical isolates, representing different genotypes in relation to alleles encoding virulence factors (MLST—multi-locus antigen sequence typing), MLVA type (multi-locus variable-number tandem repeat analysis) and PFGE group (pulsed-field gel electrophoresis) from the lungs of naive mice or mice were immunised with the commercial whole-cell pertussis vaccine, the acellular pertussis vaccine and the experimental whole-cell pertussis vaccine. Molecular data indicate that the resurgence of pertussis in populations with high vaccine coverage is associated with genomic adaptation of *B. pertussis*, to vaccine selection pressure. Pertactin-negative *B. pertussis* isolates were suspected to contribute to the reduced vaccine effectiveness. It was shown that one of the isolates used is PRN deficient. The mice were intranasally challenged with bacterial suspension containing approximately 5 × 10 ^7^ CFU/ml *B. pertussis*. The immunogenicity of the tested vaccines against PT (pertussis toxin), PRN (pertactin), FHA (filamentous haemagglutinin) and FIM (fimbriae types 2 and 3) was examined. The commercial whole-cell and acellular pertussis vaccines induced an immunity effective at eliminating the genetically different *B. pertussis* isolates from the lungs. However, the elimination of the PRN-deficient isolate from the lungs of mice vaccinated with commercial vaccines was delayed as compared to the PRN ( +) isolate, suggesting phenotypic differences with the circulating isolates and vaccine strains. The most effective vaccine was the experimental vaccine with the composition identical to that of the strains used for infection.

## Introduction

Pertussis (whooping cough), caused by *Bordetella pertussis*, is an endemic infectious disease in all countries [1]. Globally, it is estimated that in 2014, there were 24.1 million pertussis cases and 160,700 deaths from pertussis in children under 5 years of age, with periodic epidemics occurring every 2–5 years [2]. Two types of pertussis vaccines are available: whole-cell vaccines based on killed *B. pertussis* organisms and acellular pertussis vaccines which include up to 5 highly purified pertussis antigens [always pertussis toxin (PT), and a different combination of pertactin (PRN), filamentous hemagglutinin (FHA), fimbriae types 2 and 3 (FIM2 and FIM3, respectively). Due to the concerns of potential neurological side effects of the whole-cell vaccine, a less reactogenic acellular vaccine was produced in the 1980s and subsequently replaced the whole-cell pertussis vaccine in developed countries. Despite that the pertussis immunoprevention has been available for over 60 years, whooping cough is still recognised as a major public health problem. At present, the incidence of pertussis is much lower than in the pre-vaccination era, but a steady increase has been observed in countries with high vaccination coverage since the mid-1990s. In Poland, within 10 years after the introduction of pertussis vaccination in 1960, the incidence of whooping cough decreased in all age groups [3]. In the years 1982–1992, an almost 100% decrease in the average incidence of pertussis in relation to the period 1950–1960 was achieved [4]. The increase in pertussis incidence recorded in 1997 was a surprise because the pertussis vaccination programme with the national DTwP [diphtheria, tetanus and pertussis (whole-cell) vaccine] has been used consistently and the average vaccination level did not change and exceeded 95% [4]. Currently, Poland is the only EU country still using the same locally produced DTwP vaccine in children up to 2 years old [5]. Children have been continuously immunised with three primary doses and one booster dose at 2, 3–4, 5–6, and 16–18 months of age [6]. It is estimated that more than half of Polish parents choose acellular pertussis vaccines for primary vaccination today [6]. A booster dose of DTaP [diphtheria, tetanus and pertussis (acellular) vaccine]) for 6-year-old children was introduced in 2004 [5, 6]. In 2016, the number of pertussis cases in Poland was the highest in the last 20 years; according to the Department of Epidemiology and Infectious Diseases of NIPH-NIH, in 2016, there were 6856 pertussis cases (incidence 17.84), despite the fact that about 97.2% of children received primary pertussis vaccination [7]–that is why the dTap [diphtheria, tetanus and pertussis (acellular) vaccine, reduced diphtheria and pertussis antigens content] was introduced in 2016 and is given as obligatory to adolescents at the age of 14. At present, it is recommended for the booster vaccination to be given at 19 years of age, to pregnant women and to adults every 10 years. In recent years, many developed countries have reported a resurgence of pertussis despite the high vaccine coverage [8–10]. Several factors have been suggested to explain this phenomenon; the switch from wP to aP vaccines is associated with waning vaccine-induced immunity and pathogen adaptation to vaccination due to the appearance of genetically and antigenically different clinical strains from those in the vaccines’ composition [1, 11, 12].

Genetic divergence between vaccine strains and clinical *B. pertussis* isolates has been found in many developed countries, and this has been studied using pulsed-field gel electrophoresis (PFGE), multi-locus variable-number tandem repeat analysis (MLVA) and multi-locus antigen sequence typing (MAST) methods [13]. Isolates currently circulating in developed countries carry novel, non-vaccine-type alleles coding virulence factors, such as pertussis toxin S1 subunit (*ptxA*) and pertactin (*prn*). It has been shown that variation in these antigens affects vaccine efficacy in a mouse model [13–16]. Current isolates worldwide mainly are of the *ptxP3* type [14, 17]. Another mechanism of *B. pertussis* adaptation is related to increased production of pertussis toxin (Ptx) (about 1.6 times) by isolates carrying *ptxP3* allele [14]. It was shown that isolates currently circulating in developed countries express Fim3^+^ and are part of PFGE group IV [18]. In countries (among others France, Italy, Sweden, Netherlands, Finland, Norway, England) that have been using acellular vaccines for years, Prn-deficient isolates predominate [17–19].

Genetic changes observed in the *B. pertussis* isolates circulating in Poland seem to be driven slightly differently compared to elsewhere in Europe [20]. All *B. pertussis* isolates from pertussis patients in Poland after 1995 had the non-vaccine allele of the subunit S1 of pertussis toxin (*ptxA1*). In the years 1995–2013, an increase in the incidence of isolates possessing non-vaccine alleles pertactin (*prn2, prn3*), type 2 fimbriae (*fim2-2*) and pertussis toxin (*ptxC2*) was observed [20]. This distinction may reflect differences in national vaccination strategies, as (unlike the other countries) the wP vaccine is still used in Poland and no changes of vaccine strain composition have been made [20].

In the present study, our objective was to use the murine model of infection, to analyse, whether the isolates belonging to different genetic groups in relation to alleles encoding virulence factors (MLST—multi-locus antigen sequence typing), MLVA type (multi-locus variable-number tandem repeat analysis) and PFGE group (pulsed-field gel electrophoresis), colonised the mice differently, and to observe if immunity induced by the different pertussis vaccines was able to eliminate these isolates from the lungs of the vaccinated mice.Clinical isolates were chosen in line with suggestions that genetic and antigenic divergence between vaccine strains and clinical isolates may have contributed to the resurgence of pertussis [21].

## Materials and methods

### Bacterial isolates

Two *B. pertussis* isolates isolated from pertussis patients in Poland were used in the study: isolate 1330/07 (isolated in 2007 during the inter-epidemic period) and isolate 1/12 (isolated in 2012 during the epidemic period). Genotypes of isolates used for testing were determined at the NIPH-NIH with MLST (alleles encoding immunogenic virulence factors: pertussis toxin promoter (*ptxP*), pertussis toxin S1 subunit (*ptxA*), pertussis toxin S3 subunit (*ptxC*), tracheal colonisation factor A (*tcfA*) and type 2 and 3 fimbriae (*fim2*, *fim3*)), MLVA and PFGE methods [20, 22, 23]. The genetic profiles of the isolates were as follows:

strain 1330/07—*ptxA1*–*ptxC1*–*prn1*–*fim2-2*–*fim3-1*–*tcfA2*–*ptxP1*-MT70-PFGE group III and strain 1/12—*ptxA1*–*ptxC2*–*prn2*–*fim2-1*–*fim3-1*–*tcfA2*–*ptxP3-*MT27-PFGE group IV [20, 22]. These strains represented the genetic profiles dominating among isolates collected in Poland in the last decade (1/12) and in the previous decade (1330/07). Moreover, research conducted by Polak et al. [23] has shown that isolates 1/12 is PRN deficient, due to inversion of the pertactin gene promoter. *B. pertussis* isolates are stored at the NIPH-NIH in a deep-frozen state at − 70 °C.

### Vaccines used for immunisation

Commercially available paediatric DTwP vaccine (trade name DTP, produced by IBSS Biomed S.A., Cracow, Poland) and DTaP vaccine (trade name Quadracel produced by Sanofi Pasteur Limited Toronto, Ontario, Canada) were used. Experimental DTwP vaccine (produced by IBSS Biomed S.A., Cracow, Poland) was also used in the studies.

*B*. *pertussis* vaccine seed strains used for the national DTwP vaccine production were changed several times. The last change of vaccine seed strain composition took place in 1978. Since that time, the wP component of the Polish DTwP vaccine has been produced with three vaccine seed strains, 606/67, 629/65 and 186/65. All these vaccine seed strains carry the same genetic profile: *ptxA2–ptxC1–ptxP1–prn1–tcfA2–fim2-1–fim3-1*-PFGE group III [6]. A single human dose of the vaccine (SHD) is 0.5 ml and contains up to 20 billion *B. pertussis* cells. Commercial DTaP vaccine (Quadracel) contains five *B. pertussis* antigens: pertussis toxoid (20 µg), filamentous haemagglutinin (20 µg), pertactin (3 µg), and serotype-2 and serotype-3 fimbriae (5 µg). Quadracel is registered in the United States, Canada and Australia, and it is recommended for immunisation of infants from the age of 2 months and children up to the age of 6 years. In Poland, this vaccine is not routinely used. It was delivered for the request of the Polish Ministry of Health for use as a single booster dose of diphtheria, tetanus, pertussis (DTaP) and poliovirus (IPV) vaccine for children aged 6 years.

For the production of the experimental vaccine, *B. pertussis* strains were grown on H3 liquid medium with activated carbon, next were suspended in 0.9% sodium chloride solution and were inactivated by chemical (with 0.12% formaldehyde) and thermal (incubation at 25 °C for 24 h) methods. The suspension of inactivated *B. pertussis* strains was combined with the purified tetanus and diphtheria toxoids adsorbed on aluminium hydroxide. The experimental DTwP vaccine was manufactured in accordance with the pharmaceutical standard, and it is quantitatively identical with commercial DTwP vaccine. The only difference is in strains used to produce the pertussis component. The suspension of inactivated *B. pertussis* strains was produced in this case from monovalent inactivated suspensions of isolates described above (1/12 and 1330/07), mixed in a ratio 1:1.

### Animals

Five-week-old BALB/cAnNCrlCmd female mice (Institute of Experimental and Clinical Medicine of the Polish Academy of Sciences) were used for the assessment of the vaccines’ immunogenicity and for the intranasal challenge test, which allows the determination of their protective efficacy. Animal experiments were approved by the National Ethics Committee for Animal Experiments and conducted according to the Polish legislation and European Directive 2010/63/EU.

### Assessment of vaccine immunogenicity

#### Immunisation of animals

Mice were divided into 4 groups—three groups for each tested vaccine and a control group. Each group consisted of 10 animals, and each mouse was tagged for individual identification. The treatment groups were immunised intraperitoneally with 0.5 ml of the 1/20 single human dose of vaccines. The control group was vaccinated with the sterile 0.9% sodium chloride solution used for the dilution of the vaccines.

#### Blood collection

Blood for testing was collected at three time points, 30, 60 and 120 days after immunisation. Blood was collected from anaesthetised mice. For anaesthesia, a mixture of ketamine (Biowet Puławy, Poland) (0.8 mg/mouse) and xylazine (Biowet Puławy, Poland) (0.1 mg/mouse) was used. Blood was collected by cutting the plexus of the ocular vessels with a scalpel. After the collection, the blood was transferred to the incubator and kept at 37 °C ± 1 °C for 3 h. Then, it was centrifuged for 10 min at 1200 × g. The obtained serum was transferred to a new Eppendorf tube, aliquoted and stored at − 70 °C until the ELISA test was performed. Mice, after blood sampling on day 30 after immunisation, were transferred to their cages to recover from anaesthesia. After a further 30 days (60 days after immunisation), blood from the same mice was again sampled for testing. This procedure was repeated after another 60 days (120 days after immunisation). At the end of the experiment, on day 120 after immunisation, animals were euthanised by the isoflurane anaesthetic (Aerrane; Baxter, Austria) overdosing.

#### Determination of anti-PT IgG, anti-PRN IgG, anti-FHA IgG and anti-FIM IgG in mouse sera by ELISA test

The immunogenicity of vaccines was evaluated by measuring antibody titres against five pertussis antigens using ELISA method; the test was validated in-house. Maxisorp 96-well plates (NUNC, Denmark) were coated with 1 ng/µL antigen dissolved in 0.05 M carbonate buffer (Sigma Aldrich, USA). Incubation was carried out overnight in a humid chamber at 5 °C ± 3 °C. All the antigens used for coating (purified pertussis toxin, purified FHA coating antigen, purified PRN coating antigen and fimbrial agglutinogens 2, 3 coating antigens) were produced by Sanofi Pasteur, Lyon, France. The active sites were blocked with a 5% skim milk solution (AppliChem, Germany). Secondary reference standard mouse relative potency and immunogenicity (Sanofi Pasteur, France) was used to generate the antibody standard curve. Twofold standard dilutions in the range of 1/800–1/204800 for plates coated with FHA, PRN and FIM antigens and dilutions in the range of 1/1600–1/204800 for plates coated with PT antigen were used. The positive test control (mouse positive control serum; Sanofi Pasteur, Lyon, France) and the negative test control (serum from the control mice) were used to control the correctness of the test. Sera taken from mice were not pooled and each serum was tested separately. The plate was incubated for 90 min at 37 °C ± 1 °C. After washing, secondary antibody (Goat anti-mouse IgG (whole molecule) peroxidase conjugate; Sigma Aldrich, USA) was added to each well. The following conjugate dilutions were used: 1/6000 dilution for PT and FIM antigens, 1/1000 dilution for PRN antigen and 1/2000 dilution for FHA antigen. Dilutions were validated by titration of different conjugate concentrations. The conjugate was incubated for 60 min at 37 °C ± 1 °C. An OPD substrate solution (Sigma Aldrich, USA) was prepared immediately before use, at a concentration of 0.4 mg/ml in citrate buffer. Then 30% hydrogen peroxide (Sigma Aldrich, USA) was added of the resulting solution. The plate was incubated for 30 min in the dark at room temperature. The reaction was stopped by adding to each well 2 N sulfuric acid H_2_SO_4_ (DiaSorin, Italy). Optical density was read at a 490 nm wavelength using an ELISA reader (Tecan Spark 10 M).

#### Calculation of results

On the basis of the reference standard absorbance results, a standard curve was drawn by the four-parameter logistic model provided by the Labsystems Genesis v. 3.00 programme. The optical density (OD) values for the tested samples were converted into the number of units (EU/ml) in relation to the reference serum. The final results were expressed as geometric mean antibody titer (GMT) for each group of animals.

#### Acceptance criteria

The Limit of Quantification (LOQ) method was validated: for FIM antigen, it was 0.3 EU/ml, for PRN and FHA antigens, it was 1.0 EU/ml, and for the PT antigen, it was 10.0 EU/ml. LOQ was determined from the mean value plus six standard deviations (SD) of the values from the negative control wells (10 sera collected from non-immunised mice). Stimulation of a humoral response was considered to be at least higher than those expressed as LOQ for each of the antigens.

The mean optical density value obtained from the duplicate negative control samples must be less than or equal to 0.1. The value of the average optical density for the blank samples cannot be higher than 0.150. The average optical density of the positive control repeated samples must be greater than or equal to 1,000, and the coefficient of variation (CV%) of the results obtained must be less than or equal to 20%. Samples for which the optical density values obtained are in the range of the optical density values of the negative controls were treated as non-responders.

The R2 determination coefficient for the calibration curve must be at least 0.95.

The slope factor of the calibration curve must not be less than 0.98.

### Intranasal challenge test

#### Immunisation of mice

Mice were immunised with i.p. injections of 1/20 SHD of the tested vaccines. The control group was injected only with 0.9% NaCl solution.

#### Culture conditions and infection of mice

Bacteria were revived on commercially available solid media Bordetella Selective Medium (BSM) (Oxoid, Germany) at 37 °C ± 1 °C, in an atmosphere of 5% CO_2_. Strains were cultivated for 5 days. They were suspended in 1% acid casein hydrolysate. 60 days after immunisation, all mice from the experiment were intranasally challenged with 50 μl of a bacterial suspension (25 μl per nostril) containing approximately 5 × 10^7^ CFU (colony forming units) with a single *B. pertussis* strain or a mixture of each of them, in 1:1 ratio. Two hours after infection (day 0), and 7, 14 and 21 days after the challenge, four mice from each tested group were killed under anaesthesia (Aerrane; Baxter, Austria) and their lungs were removed. Individual lungs from mice were suspended in 1 ml of 1% acid casein hydrolysate and then homogenised for 5 min in the presence of metal spheres at 50 Hz (MM 301 homogeniser; Retsch, UK). Undiluted and tenfold dilutions of homogenates were seeded in an amount of 0.1 ml and grown on Bordetella selective medium. Five days later, the number of colonies were read; the number of colonies corresponding to the number of viable *B. pertussis* cells in the test sample was given as colony forming units (CFU-colony forming units) in 1 ml of lung tissue. CFUs of *B. pertussis* cultured on each agar plate were then determined, and mean CFUs were compared between mice groups at each time point. The elimination curve of *B. pertussis* strains from the lung tissue of mice belonging to the immunised and control group was determined. The CFU/ml of lung homogenate values for four animals euthanised at the same time was logarithmised (log10) and averaged. Standard deviation was determined for each time point. The lower limit of detection was 1 CFU/ml.

#### Statistical analysis

To assess the immunogenicity for each group, antibody titres and CFU values of *B. pertussis* in the lungs were compared between groups using Statistica version 10 (StatSoft, Cracow, Poland) by multi-way analysis of variance test ANOVA. In the case of obtaining significant results, the Schefee comparison procedure was applied to identify pairs of significantly influencing challenges. Statistical significance was defined as a two-tailed *p* value < 0.05.

## Results

### Immunogenicity of tested vaccines

**Fig. 1:**
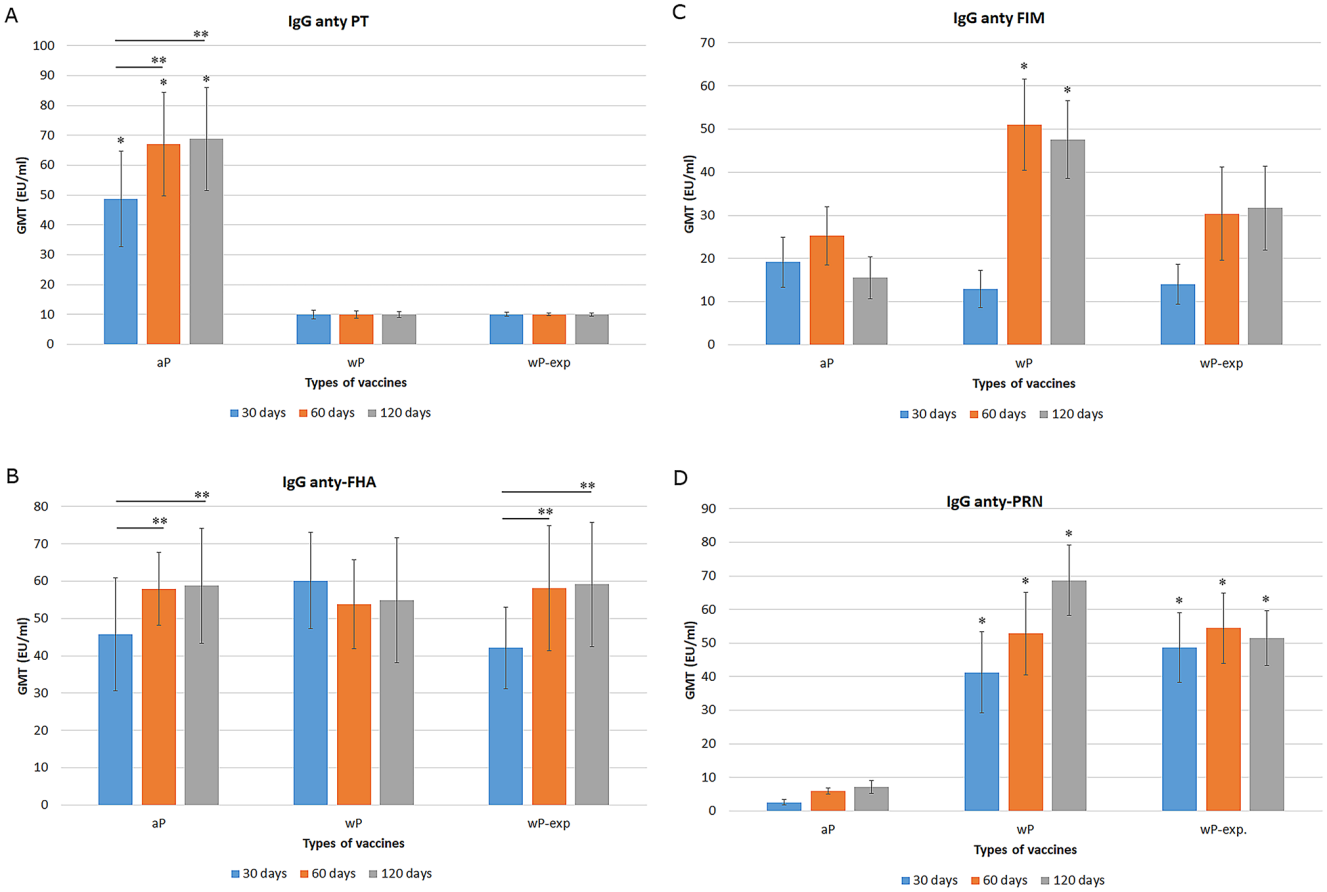
Distribution of pertussis toxin antibodies (**A**), filamentous hemagglutinin adhesion antibodies (**B**), fimbriae antibodies (**C**), pertactin antibodies (**D**) after immunisation of different types of *B.pertussis* vaccines. Results are shown as geometric mean titres (EU) per ml plus SD. ANOVA followed by Sheffe^’^s multiple comparison test was used to analyse the statistical significance between groups. Only significant differences are indicated * or * * (*p* < 0.05). * differences between different types of vaccines. ** differences between immunisation time. **A** * aP vaccine was induce significantly higher level of IgG anti-PT than wP vaccines used at any time tested after immunisation. ** Levels of IgG anti-PT increased significantly 60 and 120 days after immunisation compared to 30 days after immunisation. **B** ** From aP and wP-exp vaccines, the level of IgG anti-FHA antibodies increased significantly at 60 and 120 days after immunisation compared to the day 30. **C** * Commercial wP vaccine induced significantly higher levels of IgG anti-FIM antibodies at 60 and 120 days after immunisation compared to the other tested vaccines. **D** * IgG anti-PRN were induced at a significantly higher level by both whole-cell pertussis vaccines used than the acellular vaccine at the three time points analysed

Numerous studies have shown that antibodies to PT, FHA, PRN and FIM can provide protection against pertussis [24, 25]. Therefore, in the presented study, the humoral response after immunisation was evaluated. Using the ELISA method, we showed that the production of IgG anti-PT was induced only by the acellular pertussis vaccine. Levels of IgG anti-PT increased significantly 60 and 120 days after immunisation compared to 30 days after immunisation (Fig. 1A).

We showed that IgG anti-FHA antibodies were induced by all tested vaccines at similar levels. No statistically significant differences were observed. From aP and wP-exp vaccines, the level of antibodies increased over time and differed significantly at 60 and 120 days after immunisation compared to the measurement at day 30 (Fig. 1B).

We noted that the commercial wP vaccine induced significantly higher levels of IgG anti-FIM antibodies at 60 and 120 days after immunisation compared to the other tested vaccines. IgG anti-FIM values obtained 30 days after vaccination for all vaccines were statistically comparable; however, significantly lower IgG anti-FIM results at 120 days after vaccination were obtained using the acellular vaccine (Fig. 1C).

In our study, IgG antibodies anti-PRN were induced at a significantly higher level by both whole-cell pertussis vaccines used than the acellular vaccine at the three time points analysed. The highest levels of IgG anti-PRN antibodies were obtained 120 days after immunisation with the commercial wP vaccine (Fig. 1D).

### Intranasal challenge test

**Fig. 2:**
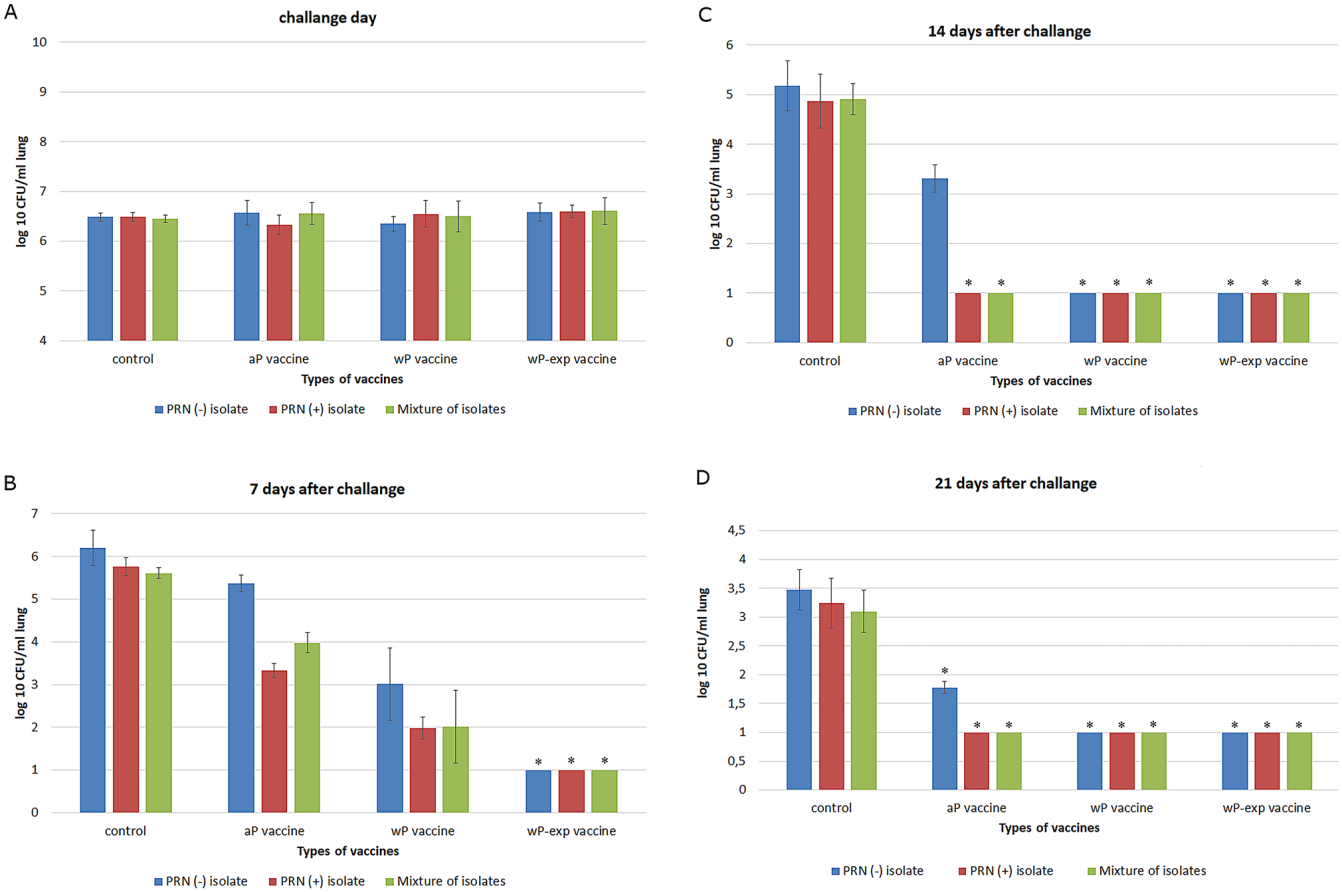
Lung clearance of *B.pertussis* isolates belonging to different genetic groups in non-immunised and immunised with aP vaccine, wP vaccine and wp-exp vaccine mice at day 0 (**A**), day 7 (**B**), day 14 (**C**) and day 21 (**D**). Intranasal challenge with 5 × 10^7^ CFU of *B. pertussis* isolates was performed in 5-week-old Balb/c control mice or in mice vaccinated with the commercial aP vaccine, commercial wP vaccine and experimental wP vaccine. Bacterial lung colonisation was measured at the indicated time points after challenge, and the results are expressed as the mean CFU per ml of lung (log10 transformed). The bacterial loads in the lungs were measured and are presented as means and standard deviations of CFU. ANOVA followed by Sheffe^’^s multiple comparison test was used to analyse the statistical significance between groups. Only significant differences between control group and immunised groups are indicated * (*p* < 0.05). **A** * No statistical differences was observed between colonisation different types of *B.pertussis* isolates in control group and immunised groups. **B** * 7 days after challenge bacterial lung colonisation in mice vaccinated wp-exp after intranasal challenge with PRN − isolate, PRN + isolate or mixture of both was significantly lower compared to unvaccinated mice. **C** * 14 days after challenge the level of colonisation with PRN − isolate, PRN + isolate or mixture of both in mice vaccinated wP-exp and wP vaccines was significantly lower compared to unvaccinated mice. *B.pertussis* PRN + isolate and mixture of both isolates were eliminated at a statistically significant level compared to the control in lungs mice vaccinated with aP vaccine also. **D** * 21 days after challenge, statistically significant lung clearance was observed in vaccinated animals compared to the control mice

In the present study, the repeatability of the intranasal challenge model was tested to validate the precision of the test in the different groups under the same experimental conditions. Repeatability of the model was verified by determination of the homogeneity of challenge suspensions and homogeneity of experimental infections. In the lungs, the average *B.pertussis* log _10_ CFU ± SD values for the control and immunised mice at day 0 reached 6.35 ± 0.53 and 6.58 ± 0.57, respectively (Fig. 2A).

The first step was to ensure that different isolates revealed a similar infection profile in mice. The colonisation of two clinical isolates PRN ( +) and PRN (−) and the mixture of it after the intranasal challenge of the mice is illustrated in Fig. 2A. They were able to multiply in the respiratory tract of naive mice. Repeatability was also confirmed by the lack of statistical differences among CFU counts obtained in experiments performed in control mice challenge with the same strains. In the control mouse group, the appropriate research model, its repeatability and bacterial viability were confirmed. The variability in bacterial count results in the lungs of control mice remained below 20% immediately after infection, which indicated that the research model was working correctly. Experiments carried out using control mice indicated that intranasally administered *B. pertussis* bacteria effectively multiply in the lungs (Fig. 2A), and a gradual natural clearance starting 1 week after challenge were observed (Fig. 2B), but not until their total eradication within 21 days (Fig. 2C, D). On the contrary, rapid lung clearance was observed in immunised animals (Fig. 2A–D).

Using the murine challenge model, we evaluated the effectiveness of the commercially wP and aP vaccines and wP experimental vaccine to eliminate the isolates that belonged to different alleles profile groups, given individually or as a mixture (Fig. 2D). As expected, significant differences between immunised and control mice were observed (*p* < 0.05) post-infection (Fig. 2D). However, the commercial vaccines have been shown to induce delayed protection against PRN (-) isolate, as shown in Fig. 2B. PRN ( +) isolate or a mixture of both isolates used were effectively eliminated by all tested vaccines by 14 days after challenge (Fig. 2C). A statistically significant difference was found in the elimination of isolates used at 7 days post-infection by the experimental vaccine compared to the commercial vaccines (Fig. 2B). The experimental vaccine was effective in eliminating the infection on day seven after infection (Fig. 2B). We have shown that commercial vaccines lead to strong protection. Seven days after challenge both wP and aP-vaccinated mice showed a reduction in bacterial load in the lungs compared to non-vaccinated mice, although not to total clearance 7 days after challenge (Fig. 2B). Commercial aP and wP vaccines demonstrated different rates of elimination of isolates used for infection. wP-vaccinated mice successfully eliminated the bacteria from the lungs by 14 days post-infection, while in aP-vaccinated mice bacteria were present even by 21 days post-infection (Fig. 2C, D).

In conclusion, we present that the incorporation of currently circulating strains into the vaccine’s composition may increase its effectiveness. We also showed that anti-PT antibodies are not necessary for promoting lung clearance of *B. pertussis* isolates.

## Discussion

Correlates of protection against whooping cough remain elusive. Characterising the antibody response to this pathogen is essential towards identifying potential correlates of protection. Evaluating pertussis vaccine-induced protection poses a problem as correlates of protection against pertussis have not been defined. A correlates of protection can be defined as an (immune) marker that statistically correlates with vaccine efficacy but is not necessarily mechanistically responsible for protection. For pertussis, a correlates of protection would ideally correlate with the inability of the bacterium to colonise the airways and would hence correlate with protection from transmission of this pathogen [26]. A number of sero-epidemiological studies [27] have shown convincingly that antibodies to *B. pertussis* antigens can be detected in the population irrespective of the local immunisation schedule, indicating that the circulation of *B. pertussis* in populations is maintained regardless of current vaccination programmes.

Antibodies to PT are induced by infection and vaccination and are used for diagnostic serology [26]. While high levels of anti-pertussis toxin (PT) have been shown to be indicative for protection against disease, no reliable threshold has been established [28]. The mechanism of antibody-mediated protection is poorly understood. Monoclonal antibodies directed against the S1 subunit of PTx have been shown to have strong protective potential in mouse models [29]. In the present study, it was shown that only the acellular vaccine induced the production of anti-PT IgG antibodies. Pertussis toxin is secreted after infection and probably the killed bacteria do not have it—that is why the wP vaccines do not activate antibodies against it. However, in our study, despite the lack of seroconversion to the PT antigen, whole-cell vaccines proved to be more effective in eliminating bacteria from the lungs of immunised mice than acellular vaccines. Most likely, anti-PT IgG antibodies are not necessary to eliminate bacteria from the lungs of infected mice. This is in line with the clinical studies that have not confirmed the correlation of anti-PT antibody titers with the efficacy of the wP vaccine in preventing pertussis [30, 31]. Convincing evidence exists, proving that anti-PT antibodies provide passive protection against pertussis, but do not participate in the primary response [32]. On the other hand, in Denmark, they have been using a PT-only acellular vaccine for 17 years, and vaccine failures have not increased [32].

In mouse models, antibodies to FHA were the least protective of all vaccine antigens against pertussis challenge, but did boost the protection conferred by PT [33]. The importance of anti-FHA antibody responses in reducing the risk of whooping cough is ambiguous [34]. In our study, none of the tested vaccines induced a significantly higher level of anti-FHA IgG compared to the others, despite there were differences in the elimination of bacteria from the lungs. This may be consistent with the assumption that FHA is the least important antigen providing protection against illness in children [35]. This observation was also confirmed by in vivo studies in which it was observed that anti-FHA antibodies alone did not protect mice against intracerebral administration of *B. pertussis* strain [36, 37] and that they played the smallest role in protection against nasal infection [32]. In the presence of anti-PRN, anti-PT and anti-FIM antibodies, the presence of anti-FHA antibodies did not contribute to increased protection [38]. Passive administration of high titre anti-PT and anti-PRN sera provided effective protection against *B. pertussis* infection in experimental animals, and the level of protection was higher than when only high titre of anti-FHA sera was passively administered [346. The lack of widespread occurrence of non-FHA-producing strains in the era of acellular vaccines suggests that this is not critical for pathogenicity of the *B. pertussis* virulence factor. Previous studies by another team suggest that FHA should not be included in a new formulation of vaccines [32].

In vitro, antibodies to FIM2 and FIM3 inhibited bacterial attachment to cells [39] and in a tracheal organ culture model, mutant strains lacking FIM had reduced adherence compared with a wild-type strain [40]. In mice, antibodies against FIM2/3 increased protection and reduced colonisation after intranasal challenge [41]. FIM antibodies may also booster the immunity to pertussis by promoting opsonophagocytosis [39]. These study observed that level of anti-FIM IgG antibodies 30 days after vaccination for all three vaccines tested was statistically comparable. As measured after 60 and 120 days, anti-FIM IgG values for the commercial wP vaccine were significantly higher than for the other two vaccines. The differences in immunogenicity of the two tested whole-cell vaccines from FIM antigen may be due to differences in the strains used for their production. FIM 2 and FIM 3 production is not stable; the World Health Organisation (WHO) recommends, therefore, that both Fim2- and Fim3-producing strains should be used for the preparation of whole-cell vaccines [42] which may be associated with the expression of fimbriae, depending on the growing conditions of the strains. However, strains that express predominantly FIM2 can also express FIM3 during infection [43]. Given the involvement of the fimbriae in protection, fimbrial production must be sufficiently stable in the live *B. pertussis* vaccine strains to induce immune responses to these antigens. The stability of the Fim2 and Fim3 production deserves particular attention since phase variation from one serotype to another has been described [44]. The largest decrease in anti-FIM IgG levels was observed between 60 and 120 days for aP vaccine. In another study, it was showed that in mice immunised with pertussis vaccines, the level of antibodies against PT, FHA and PRN antigens increases very slowly, but then decreases rapidly and is undetectable after 6–9 months from vaccination [45].

Especially relevant are the results of two independent field trials, which revealed a correlation between pertactin antibodies and clinical protection [32, 46]. The results of our study showed that whole-cell vaccines induced significantly higher levels of anti-PRN IgG as compared to the acellular vaccine. The hypothesis may be drawn that PRN plays an important role in bacterial invasion, and anti-PRN antibodies are crucial in the first line of defence because differences in the rate of elimination of the PRN (−) strain were observed compared to the PRN ( +) strain after immunisation with the commercial vaccines tested. This may be correlated with the production of protective anti-PRN antibodies. Mice that possess anti-PRN antibodies are highly resistant to nasal infection with a virulent *B. pertussis* strain [47]. A study on the efficacy of acellular pertussis vaccine carried out in Germany showed that with high anti-pertactin antibody values, other antibody titers were not important in preventing disease [48]. There have been reports suggesting that mainly anti-PT and anti-PRN antibodies are necessary to provide protection [34, 49]. In trials in Erlangen and in Sweden, it was found that antibody to PRN and FIM were most important and antibody to FHA did not contribute to efficacy. They also both noted a curious finding, if you had high values to PT and FIM, you were less well protected than if you had a low value to PT [35]. More recent studies by Weiss et al. suggest that this could be a blocking effect of excessive PT on PRN and FIM [50]. In other studies conducted by King et al. [45] has proven that presence of high concentrations of multiple antigens may minimise the effect of variation in pertactin. Some data suggested that PT is a critical vaccine component, but too much may be detrimental [32]. Since PRN is an outer membrane protein, contrary to PT that is secreted proteins, it could be more sensitive to herd immunity [19]. Improvement of the acellular pertussis vaccines may be worthwhile.

Although no specific level of antibody against antigens of *B. pertussis* has been convincingly shown to confer protection against the disease, the prevalence of these antibodies at different ages can be used as an index of the exposure to pertussis antigens [26, 51]. In addition to the quantity, the quality and functionality of antibodies should also be evaluated in future [53]. One of the functions of antibodies is to facilitate the uptake of bacteria via Fc-γ-receptor-mediated opsonophagocytosis by, for example, neutrophils which play an important role in clearing *B. pertussis* during infection and, thus, in preventing colonisation [52]. During an infection with *B. pertussis*, neutrophils infiltrate the lung where they are important for the clearance of this respiratory pathogen [52]. Some authors propose antibody-mediated opsonophagocytosis of *B. pertussis* as a possible correlates of protection against pertussis [26, 53]. Anti-pertactin antibodies were found to be crucial for *B. pertussis* phagocytosis [54]. The study conducted by Hellwig et al. [54] provides a rational basis for the observed correlation between protection against pertussis and antibodies to PRN and shows that such antibodies are crucial for phagocytosis of *B. pertussis* by immune cells. After depletion of antibodies to PRN, the ability of the serum to induce phagocytosis was nearly absent. It remains unknown whether specific antibodies generated upon infection and vaccination are similar in functionality. Interestingly, FHA- and PT-specific antibodies did not show an increased avidity in the recovered pertussis patients suggesting that perhaps high antibody avidity for these antigens is not necessarily important for opsonophagocytosis of *B. pertussis* [54].

The murine intranasal challenge model has been shown previously to be correlated with vaccine efficacy in humans [33, 51, 55, 56]. Using the murine intranasal challenge model, it was shown that immunisation of mice prior to infection with PRN-deficient isolates with wP and aP vaccines lead to an early clearance of bacteria from the respiratory tract of mice as compared to the non-vaccinated animals, but the antigen-specific whole-cell vaccine (wP-exp) was characterised by the best efficacy and the rate of elimination of *B. pertussis* strains. In the analysis carried out in the study, different strain elimination rates were observed, depending on the type of vaccine used for immunisation. Our studies have shown that immunisation with aP vaccine provides protection, but clearance of *B. pertussis* from the lungs is slower in comparison to mice immunised with the wP vaccine. It has been shown that the elimination of bacteria from the lungs of mice immunised with aP vaccine begins between 7 and 14 days after infection, and in the case of mice immunised with wP vaccine, this elimination occurs approximately 7 days earlier. This is confirmed by the studies performed in the mouse model [57] and baboon model [58] that shown that only whole-cell vaccines reduce the colonisation with *B. pertussis*. This fact most probably confirms that vaccines with whole-cell and acellular pertussis component induce different effector mechanisms of the immune response [58–60].

Isolates with vaccine-type pertactin were found in lower frequencies in vaccinated children compared to non-vaccinated children, suggesting that variation in pertactin affects vaccine efficacy [61]. Our study did not show that the PRN (-) strain had a better ability to adapt in the non-vaccinated mice. The rate of multiplication of each strain used for infection, together and separately, in the lungs of control mice was very similar. The elimination rate of the strain mixture was comparable to that of each strain administered to mice separately. The results reported by another research team also showed that the lack of pertactin production did not affect the increased virulence of *B. pertussis* [62]. *B. pertussis* strains not producing pertactin are increasingly isolated from people with pertussis disease symptoms [18, 63, 64]. There are reports proving that PRN (−) strains do not cause stronger clinical symptoms in infants less than 6 months old, from whom they were isolated [17, 19]. However, there are also reports demonstrating that PRN (−) isolates show greater virulence in populations immunised with aP vaccines [65]. This observation has also been suggested recently by US researchers [40]. PRN-producing strains more often infected younger patients, and in the course of the disease, the acute phase was more common. Patients who received at least one dose of acellular pertussis vaccine were twice as likely to be infected with PRN (−) strain compared to unvaccinated ones. It is suggested that the loss of PRN antigen production in these strains may bring a selective advantage by reducing the severity of clinical symptoms, which prolonged the diagnosis and extends the transmission time of bacteria to other susceptible persons [66, 67]. King et al. [45] and Bottero et al. [68] presented evidence to suggest that polymorphism of *ptxA* or *prn* can affect vaccine efficacy for clinical isolates. In contrast, Boursaux-Eude et al. showed that commercial vaccines in France were effective against circulating *B. pertussis* isolates, regardless of *ptxA* and *prn* alleles [69]. Our data suggest that the current commercial vaccines used in this study would be effective against circulating strains without regard to *prn* alleles. However, if antigen mismatch shortens the duration of immunity, strains with antigens that do not match those in the vaccine may play an important role in increasing pertussis cases, especially in adolescent and adult populations whose immunity to pertussis has waned [69].

From the results presented above, it is clear that the efficacy of vaccine increases directly with the number of antigens in the vaccine [46]. It should be noted that wP vaccines contain about 3000 *B. pertussis* proteins and antibody to many of these contribute to protection [70].

wP vaccination induces a broad immune response against many bacterial antigens and virulence factors since they are composed of killed entire bacteria. aP vaccines are composed of between one and five purified detoxified antigens and consequently induce immunity against only a few bacterial proteins involved in the virulence of the bacterium. *B. pertussis* produces many virulence factors, and clinical isolates may differ in other properties. That is the reason it will be important to perform not only genomic studies but also proteomic studies on the isolates to analyse at which level are located the differences between them. Overall, it seems most probable that no single correlate of protection exists and that antibodies to many antigens in differing amounts, probably in conjunction with cell-mediated immunity, confer protection against symptomatic reinfection of *B.pertussis* [71].
